# The Development and Performance of Knitted Cool Fabric Based on Ultra-High Molecular Weight Polyethylene

**DOI:** 10.3390/polym16030325

**Published:** 2024-01-25

**Authors:** Yajie Zhao, Zhijia Dong, Haijun He, Honglian Cong

**Affiliations:** Engineering Research Center of Knitting Technology, Ministry of Education, Jiangnan University, Wuxi 214122, China; zyj15505295676@126.com (Y.Z.); dongzj0921@163.com (Z.D.); hhj@jiangnan.edu.cn (H.H.)

**Keywords:** knitting, cool fiber, UHMWPE, comfortable with heat and humidity, cooling sensation

## Abstract

In order to withstand high-temperature environments, ultra-high molecular weight polyethylene (UHMWPE) fibers with cooling properties are being increasingly used in personal thermal management textiles during the summer. However, there is relatively little research on its combination with knitting. In this paper, we combine UHMWPE fiber and knitting structure to investigate the impact of varying UHMWPE fiber content and different knitting structures on the heat and humidity comfort as well as the cooling properties of fabrics. For this purpose, five kinds of different proportions of UHMWPE and polyamide yarn preparation, as well as five kinds of knitted tissue structures based on woven tissue were designed to weave 25 knitted fabrics. The air permeability, moisture permeability, moisture absorption and humidity conduction, thermal property, and contact cool feeling property of the fabrics were tested. Then, orthogonal analysis and correlation analysis were used to statistically evaluate the properties of the fabrics statistically. The results show that as the UHMWPE content increases, the air permeability, heat conductivity, and contact cool feeling property of the fabrics improve. The moisture permeability, moisture absorption and humidity conductivity of fabrics containing UHMWPE are superior to those containing only polyamide. The air permeability, moisture permeability, and thermal conductivity of the fabrics formed by the tuck plating organization are superior to those of the flat needle plating and float wire plating organization. The fabric formed by 2 separate 2 float wire organization has the best moisture absorption, humidity conduction, contact cool feeling property.

## 1. Introduction

Recently, with the continuous intensification of the greenhouse effect, the ambient temperature in summer has become hotter, and the utilization rate of refrigeration equipment has soared. However, the energy consumption and heat generated by refrigeration equipment contributes to the greenhouse effect, creating a vicious cycle [1]. The application of cool fabrics can alleviate this phenomenon to a certain extent. Cool fabric, quite literally, is the type of fabric that creates a cooling sensation when it comes into contact with the skin. When in direct contact with human skin, this material can quickly absorb and conduct sweat on the skin’s surface, and rapidly dissipate the heat generated by the body’s hot environment, keeping the skin dry and cool upon contact. It can keep you cool and comfortable for a long time and plays a role in regulating the microclimate on the surface of human skin [2,3]. Cool feeling fabrics can be divided into passive heat dissipation textiles, active cooling textiles, temperature and moisture responsive textiles from the principle of development [4,5].

At present, there are mainly three methods to realize the coolness of fabrics, namely, the application of the fiber with coolness itself or the physicochemical modification of coolness fiber, the fabric structure design, and finishing of fabrics with coolness additives [6,7]. Common fibers to achieve fabric coolness [8] include fibers with high thermal conductivity when spinning, such as jade powder, mica powder and other fibers with high thermal conductivity minerals [9,10,11], as well as fibers with shaped cross sections designed by melt spinning [12,13,14]. In terms of organizational structure, fabrics primarily achieve cooling through pore heat dissipation and water conduction heat dissipation. The lower the interweaving of yarn in the tissue unit of the fabrics, the higher the porosity, the better the permeability and heat performance of the fabrics [15]. Under the same conditions, the smaller the tightness and thickness of the fabric, the better the cooling performance [16]. The looped knitted fabric has better air permeability and dynamic cooling than the floating knitted fabric [17]. The post-finishing of cooling additives [18] is a method used to add additives containing menthol, xylitol, and other functions of moisture absorption and heat absorption in the process of fabric finishing, so that the fabric has a cool feeling. However, when using the cool aid to arrange the fabric, the durability of the cool fabric cannot be guaranteed, and the process is relatively complex. Thus, the application of cool fibers and the design of the structure are the best ways to achieve the cool feeling of fabrics.

Most of the existing research on cool fabrics focuses on adding high thermal conductivity substances into the spinning stock solution or changing the shape of spinneret holes during spinning to produce fibers with high thermal conductivity or irregular cross sections, so as to prepare cool fabrics [19,20]. However, this method is not only complex and challenging to implement, but also the inclusion of high thermal conductivity substances will diminish the mechanical properties of the fiber and compromise the durability of the fabric. In addition, fibers with high hygroscopic properties are used to prepare cool fabrics [21]. However, such fabrics have limited cold contact, and may cause a stuffy feeling after a long time. The ultra-high molecular weight polyethylene (UHMWPE) fiber does not require the addition of high thermal conductivity minerals or the design of irregular cross sections during the spinning process. Its high thermal conductivity, high infrared transmittance, and smooth surface accelerate the conduction of heat and water, achieving a cooling effect [22,23]. This process does not alter the original performance of the fiber, making it a superior material for weaving cool fabric. Knitted fabrics are superior to woven fabrics in terms of flexibility, ductility, and breathability. However, there is relatively little research on combining UHMWPE with knitting technology.

In this paper, we utilized UHMWPE filament with high thermal conductivity, combined with polyamide filament and polyamide/spandex covered yarn, to prepare five types of yarns with varying UHMWPE content. Additionally, we designed and wove 25 different fabrics using five different knitting structures based on the plating stitch. The aim of this study was to examine the impact of varying UHMWPE content and different fabric structures on the thermal and wet-related properties of fabrics, with the goal of facilitating the development of cooling fabrics.

## 2. Materials and Methods

### 2.1. The Materials

Ultra-high molecular weight polyethylene (UHMWPE) fiber, also known as high-strength and high-modulus polyethylene fiber, has good chemical stability, cut resistance, hydrophobicity, and coolness. It is mostly used in medical, industrial, and national defense sectors. However, due to ongoing advancements in industrial technology, UHMWPE is increasingly finding applications in the textile industry [24]. Studies have found that the thermal conductivity of common fibers ranges from 0.042 to 0.337 W/(m·°C), as shown in Table 1. In contrast, the thermal conductivity of UHMWPE fiber ranges from 0.3 to 0.5 W/(m·°C), demonstrating excellent thermal conductivity [25]. The fabric developed with this fiber has excellent cooling properties. Studies have found that it is possible to interweav UHMWPE fiber with other functional fibers [26,27]. Three types of yarn were selected for fabric knitting: 8.33 tex UHMWPE filament, 7.78 tex polyamide filament, and 8.89 tex polyamide/spandex covered yarn (consisting of 5.56 tex polyamide clad with 3.33 tex spandex).

### 2.2. Fabric Structure Design and Preparation

**Figure 1:**
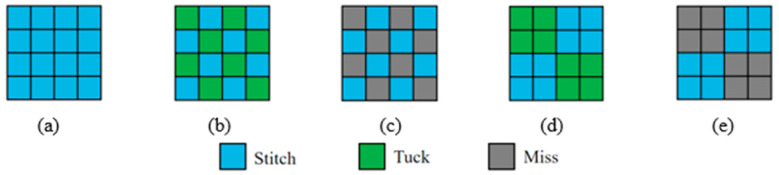
Fabric structure design diagrams; (**a**) flat needle plating organization; (**b**) single needle single column set circle add yarn organization; (**c**) 1 separate 1 float wire organization; (**d**) double needle and double column set circle to add yarn organization; (**e**) 2 separate 2 float wire organization.

**Figure 2:**
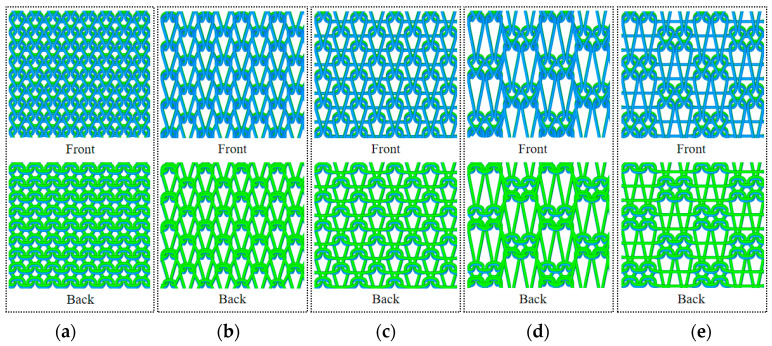
Fabric coil structure diagrams; (**a**) flat needle plating organization; (**b**) single needle single column set circle add yarn organization; (**c**) 1 separate 1 float wire organization; (**d**) double needle and double column set circle to add yarn organization; (**e**) 2 separate 2 float wire organization. The light blue lines represent the upper yarn of each add yarn organization, and the light green lines represent the ground yarn of each add yarn organization.

The varying fabric structures not only impact the surface characteristics of the fabric, such as smoothness and pore size, but also influence the intrinsic properties of the fabric, such as thermal and moisture comfort, at a deeper level. Five different organizational structures were designed based on the knitting fabric: flat needle plating organization (a), single needle single column set circle add yarn organization (b), 1 separate 1 float wire organization (c), double needle and double column set circle to add yarn organization (d), and 2 separate 2 float wire organization (e). The structural design diagram is depicted in Figure 1. The diagram of the coil structure is shown in Figure 2. 

**Figure 3:**
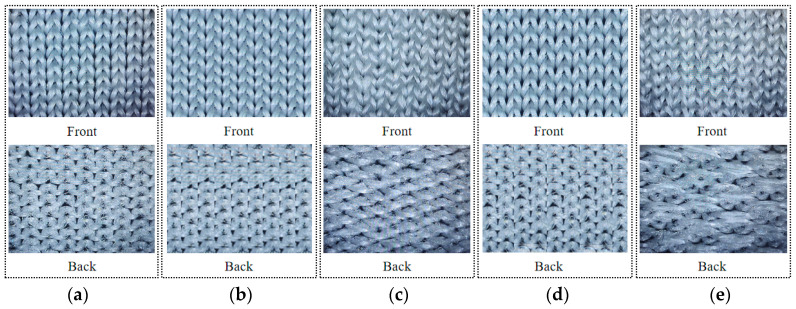
Fabric physical pictures; (**a**) flat needle plating organization; (**b**) single needle single column set circle add yarn organization; (**c**) 1 separate 1 float wire organization; (**d**) double needle and double column set circle to add yarn organization; (**e**) 2 separate 2 float wire organization.

The Saint-toni SM8-TOP2 MP2 seamless underwear machine is utilized for fabric knitting. This equipment has a machine number of E28 needles/inch and a total of 1344 needles. It is equipped with an 8-way yarn feeding system, with each way having 8 yarn nozzles and 2 needle selectors [28,29,30]. The physical pictures of the fabrics woven using the five knitting structures mentioned above are shown in Figure 3.

To comprehensively assess the performance of UHMWPE fabric, we selected UHMWPE filament and polyamide filament as the raw materials for the upper yarn, and polyamide/spandex covered yarn for the ground yarn. By altering the number of yarn feeding routes for the two types of upper yarn, five different upper yarn configurations have been designed for fabric knitting. These configurations are as follows: 8U0N, where all upper yarns from the 1st to the 8th are UHMWPE filament; 6U2N, where except for the upper yarn of the 1st and 3rd routes, the upper yarn of the other 6 routes are UHMWPE filament; 4U4N, where the 4 odd-numbered upper yarns are polyamide filament, and the 4 even-numbered upper yarns are UHMWPE filament; 2U6N, where except for the upper yarns of the 2nd and 4th routes, which are UHMWPE filament, the upper yarns of the other 6 routes are polyamide filament; 0U8N, where all upper yarns from the 1st to the 8th are polyamide filament. In the preparation of these five yarns, the proportion of UHMWPE filament in the upper yarn (*P_U_*) was determined to be 100%, 75%, 50%, 25%, and 0%. The factors and levels of the samples are presented in Table 2. The properties of fabrics were compared and analyzed primarily based on two factors: upper yarn configuration and organizational structure. The study comprehensively considered 5 levels of upper yarn configuration and 5 levels of organizational structure, resulting in the weaving of 25 groups of experimental samples (5 × 5 = 25). The basic specifications of the samples are presented in Table 3.

### 2.3. Fabric Property Test

#### 2.3.1. Air Permeability

The air permeability of a fabric refers to the ability of air to pass vertically through a specified test area of the fabric under a specific pressure difference within a given unit of time [31,32]. According to GB/T 5453-1997 “Textiles—Determination of the permeability of fabrics to air” [33], the air permeability of the fabric was tested using the YG461E-III automatic air permeability meter from Ningbo Textile Instrument Factory (Ningbo, China). The test result was measured by the air permeability ratio (*Q*), with higher ratios indicating better air permeability. The test pressure difference set during the testing process is 100 Pa, with a test area of 20 cm^2^. Ten different sections of each fabric are selected to measure its air permeability, and the average value is calculated for each section.

#### 2.3.2. Moisture Permeability

The moisture permeability of fabric, also known as water vapor permeability, refers to the fabric’s ability to allow water vapor to pass through, specifically the fabric’s permeability to gaseous water [34]. According to GB/T 12704.1-2009 “Textiles—Test method for water-vapor transmission of fabrics—Part 1: Desiccant method” [35], the moisture permeability of the fabric was tested using the YG601H-II type computerized fabric permeability meter from Ningbo Textile Instrument Factory (Ningbo, China). The test result was measured by the moisture permeability ratio (*WVT*). The calculation formula is Formula (1). The higher the value, the greater the moisture permeability. Prepare three round samples of each fabric with a diameter of approximately 7 cm, and then average the results.

(1) $$WVT=\frac{24\times \Delta m}{S\times t}$$

where: *WVT* represents the moisture permeability ratio of the sample, measured in [g·(m^2^·24 h)^−1^]; Δ*m* is the difference between two weighing masses of the same test assembly, measured in grams; *S* denotes the effective experimental area of the sample, with a value of 0.00283 m^2^ for this experiment; *t* stands for the test time, measured in hours. 

#### 2.3.3. Moisture Absorption and Humidity Conduction

The moisture absorption property of the fabric refers to its ability to absorb liquid water, while the humidity conduction property of the fabric pertains to its ability to conduct liquid water [36]. According to GB/T 21655.2-2009 “Textiles—Evaluation of absorption and quick-drying Part 2: Method for moisture management tests”, [37] the Q290 liquid moisture management analyzer (Standard Group (Hong Kong) Limited, China) is used to test the fabric’s wetting time (upper *T*1, lower *T*2), absorption speed (upper *A*1, lower *A*2), and accumulative one-way transport capacity (*O*) to collectively characterize the moisture absorption and humidity conduction of the fabric. Before the test, a NaCl solution with a mass concentration of 9 g/L should be prepared to simulate human sweat. After preparing the instrument, five square samples measuring 8 cm × 8 cm of each fabric are tested sequentially. During the test, the fabric is positioned with the skin side facing up, and the average value is then recorded.

#### 2.3.4. Thermal Property

The thermal conductivity of fabrics, also known as heat transfer, refers to the ability of fabrics to conduct heat [38]. According to GB/T 11048-2008 “Textiles—Physiological effects—Measurement of thermal and water-vapor resistance under steady-state conditions”, [39] the thermal properties of fabrics were tested using the YG606G thermal resistance and wet resistance tester manufactured by Ningbo Textile Instrument Factory (Ningbo, China). The test results were measured using three indexes: thermal resistance (*R_t_*), warmth retention ratio (*W_t_*), and heat transfer coefficient (*K_t_*). The lower the thermal resistance and the higher the warmth retention ratio, the better the heat transfer coefficient, and thus the better the heat transfer properties of the fabric. Prepare three square samples of 35 cm × 35 cm for each type of fabric. After the machine has been preheated, place the samples on the test board one by one for testing (with the skin side of the fabric facing the test board, i.e., the skin side facing downward), and then calculate the average value. 

#### 2.3.5. Contact Cool Feeling Property

The contact cool feeling property of fabric refers to the phenomenon where the skin comes into contact with fabric that is cooler than the skin’s temperature. This results in rapid heat exchange from the skin surface to the fabric, causing an instantaneous drop in skin surface temperature. This change is then relayed to the brain through the thermosensitive nerve endings in the skin, creating a sensation of coolness [40]. According to GB/T 35263-2017 “Textiles—Testing and Evaluation for Instant Contact Cool Feeling”, [41] the contact cool feeling property of the fabric was tested using the MB291 Fabric Cooling Performance Tester (Quanzhou Meibang Instrument, Quanzhou, China). The test result was measured by the contact cool feeling coefficient *q*_max_. The higher the contact cold feeling coefficient, the more intense the sensation of coolness the skin experiences from the fabric. Five square samples, each measuring 20 cm × 20 cm, of each fabric were prepared and tested sequentially, and the results were averaged.

## 3. Results and Discussion

The study investigated the air permeability, moisture permeability, moisture absorption and humidity conduction, thermal property and contact cool feeling property of 25 types of knitted fabrics. The performance test results are presented in Table 4. The standard deviation values for each test result are shown in Table 5. It can be seen from Table 5 that the data of each performance index are more reliable. Orthogonal test analysis and correlation analysis were conducted on the test results to investigate the impact of various upper yarn configurations and organizational structures on fabric properties, as well as the factors influencing these properties.

### 3.1. Air Permeability

The air permeability of fabric is influenced by various factors, with yarn type and structure being the primary ones [42]. The orthogonal test results for the air permeability test in Table 4 are presented in Table 6. It can be observed that the R-value of factor A is smaller than that of factor B, indicating that the fabric structure has a greater impact on the sample’s air permeability. This may be because the impact of the inter-yarn porosity on the air permeability of the fabric is slightly greater than that of the inter-fiber porosity. In addition, it can also be observed that achieving the best weaving scheme for fabric air permeability is A_1_B_4_.

**Figure 4:**
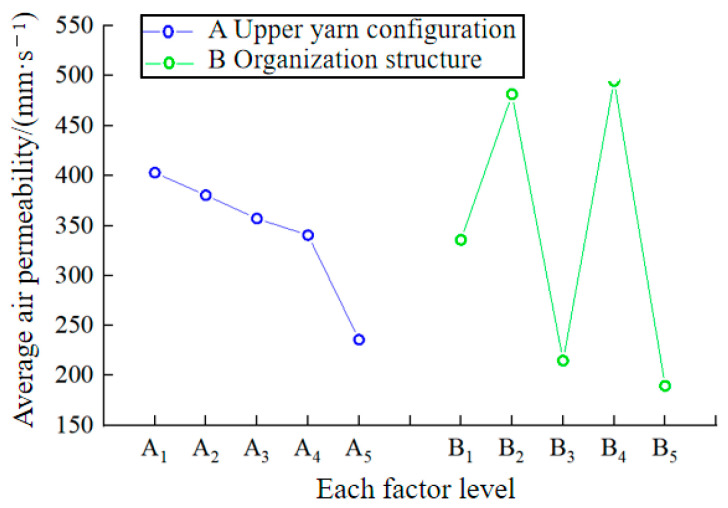
The relationship between the level of each factor and the average air permeability of the sample.

To more intuitively study the influence of the two factors and five levels of the design on the air permeability of the sample, the relationship diagram in Figure 4 was created by plotting the level of each factor on the horizontal axis and the average air permeability ( $\bar{K}{\;}_{i}$ value) of the fabric under each factor level on the vertical axis. It can be observed from the figure that the influence of each level in the upper yarn configuration of factor A on the air permeability of the fabrics is ranked as follows: A_1_ > A_2_ > A_3_ > A_4_ > A_5_. This indicates that as the proportion of UHMWPE filament in the upper yarn decreases, the air permeability ratio of the fabric also decreases, leading to a deterioration in air permeability. This may be due to the fact that as the proportion of UHMWPE filament decreases and the proportion of polyamide filament increases within the same structure, the mass per unit area and thickness of the fabric increase. Consequently, the passage of air through the fabric decreases and extends, leading to a reduction in air permeability. The order of influence degree of each level in the organizational structure of factor B on the air permeability of the fabrics is as follows: B_4_ > B_2_ > B_1_ > B_3_ > B_5_. This indicates that the air permeability of the fabric formed by the tuck plating organization is the best, followed by the flat needle plating organization, and the float wire plating organization is the worst. This may be because under the preparation of the same yarn, the mass per unit area of the fabric formed by the tuck plating organization is smaller than that of the fabric formed by the flat needle plating organization and the float wire plating organization. Additionally, the tuck plating organization can create a hole effect on the fabric surface, allowing air to pass through more easily, resulting in better air permeability.

According to the orthogonal analysis results above, the primary factor influencing the air permeability of the fabric is its structure. Different structures will exhibit distinct variations in mass per unit area and thickness. The correlation analysis between air permeability and the specification parameters of the fabrics was conducted, and the results are shown in Table 7. It can be seen that the air permeability of the fabrics is highly correlated with the mass per unit area and thickness of the fabric (0.8 < |r| ≤ 1). This correlation is negative, indicating that fabrics with smaller mass per unit area and thinner thickness have greater air permeability and better air permeability. There was a weak positive correlation between the proportion of UHMWPE filament in the upper yarn configuration (0.3 < |r| ≤ 0.5), and the fabric’s air permeability. A larger proportion of UHMWPE filament in the upper yarn configuration was associated with better air permeability.

### 3.2. Moisture Permeability

The primary factors influencing the moisture permeability of fabrics include fiber characteristics, yarn characteristics, and fabric structure characteristics [43]. The orthogonal test results for the moisture permeability test in Table 4 are presented in Table 8. It can be seen that the upper yarn configuration of the fabric had a significant impact on the moisture permeability of the sample. In addition, it can also be seen from the table that the optimal weaving scheme to achieve the best fabric moisture permeability is A_3_B_4_.

**Figure 5:**
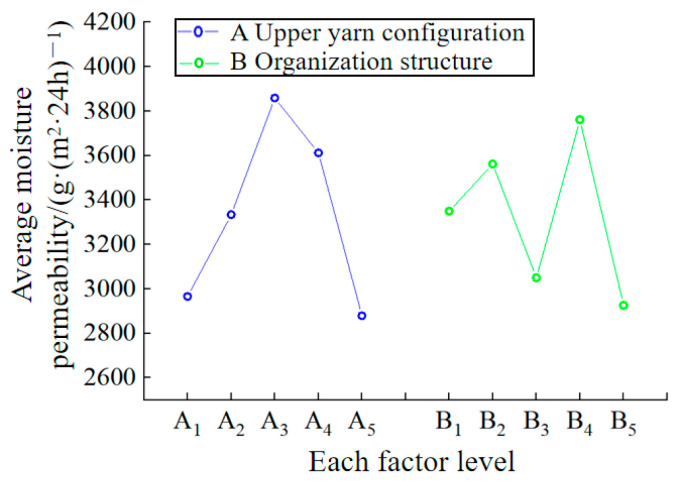
The relationship between the level of each factor and the average moisture permeability of the sample.

To intuitively study the influence of two factors and five levels on the moisture permeability of the sample, the factor level is taken as the x-axis, and the average permeability of the fabric under the factor level ( $\bar{K}{\;}_{i}$ value) as the y-axis. The relationship diagram of each factor level and average moisture permeability is shown in Figure 5. It can be observed from the figure that the influence of each level in the upper yarn configuration of factor A on the moisture permeability of the fabrics is ranked as follows: A_3_ > A_4_ > A_2_ > A_1_ > A_5_. This indicates that the moisture permeability of the fabric is optimal when the ratio of the number of yarn routes between the UHMWPE filament and the polyamide filament in the upper yarn configuration is equal. This is because, under the same structure, the polyamide fiber has better moisture absorption than UHMWPE, and the fabric containing polyamide fiber has better moisture permeability than pure UHMWPE fabric. However, due to the continuous absorption of moisture by the polyamide fiber, the fiber will expand, causing the gap between the fibers to become smaller, resulting in poor moisture permeability of pure polyamide fabric. The order of influence of each level in the organizational structure of factor B on the moisture permeability of the fabrics is as follows: B_4_ > B_2_ > B_1_ > B_3_ > B_5_. This indicates that the moisture permeability of the fabric formed by the tuck plating organization is better, while the float wire plating organization is the worst. This is because, under the same yarn preparation, the fabric formed by the tuck plating organization has a larger aperture, resulting in a larger specific surface area, more water transfer channels, and better moisture permeability.

According to the orthogonal analysis results above, a correlation analysis was conducted between the fabric’s moisture permeability and its specification parameters, as well as other performance indicators. The results are presented in Table 9. It is evident that the moisture permeability of the fabrics is significantly correlated with the surface density and air permeability of the fabrics (0.5 < |r| ≤ 0.8) and has a low correlation with the thickness (0.3 < |r| ≤ 0.5). It shows that fabric with a low quality per unit area and good air permeability has relatively good moisture permeability. There was no correlation between the proportion of UHMWPE filament in the upper yarn configuration (0 < |r| ≤ 0.3).

### 3.3. Moisture Absorption and Humidity Conduction

The factors that primarily affect the moisture absorption and humidity conduction of fabrics include the hydrophilicity of fibers and the fabric structure [44,45,46]. The shorter the soaking time, the higher the water absorption rate and the greater the one-way transfer index, leading to improved moisture absorption and humidity conduction of the fabric. According to the evaluation method outlined in GB/T 21655.2-2009 for assessing the moisture absorption of the fabric in Table 4, it is evident that all 25 fabrics exhibit excellent moisture absorption. When the UHMWPE fiber content exceeds that of the polyamide, the moisture conductivity of the fabrics is improved. The orthogonal test was conducted following the standardization of the test results for the positive indicators (soaking time) and negative indicators (water absorption rate and one-way transfer index) of the fabrics in Table 4. The analysis results are presented in Table 10. The comprehensive hygroscopic moisture guide index *C* (the sum of data after standardized processing of *A*1, *A*2, *T*1, *T*2, and *O*) was used to comprehensively represent the moisture absorption and humidity conduction of the fabric. It can be observed that A_2_B_5_ is the optimal weaving pattern for enhancing the fabric’s moisture absorption and humidity conduction.

**Figure 6:**
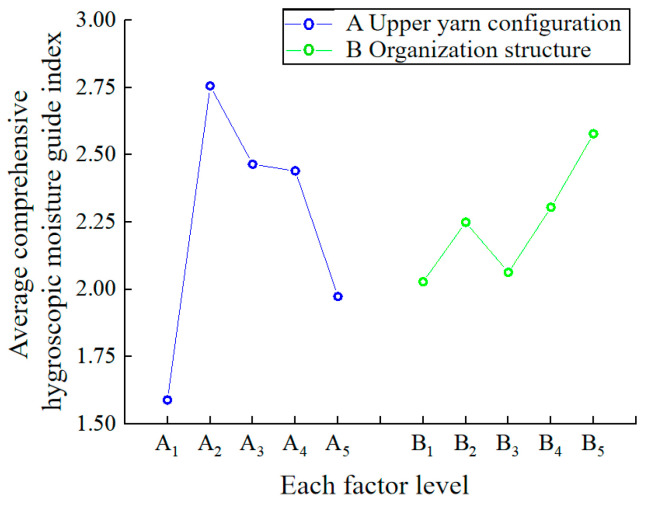
The relationship of each factor level and the average comprehensive hygroscopic moisture guide index.

To intuitively study the influence of two factors and five levels on the moisture absorption and humidity conduction of the sample, each factor level was used as the horizontal coordinate, and the average comprehensive hygroscopic moisture guide index of the fabric under the factor level ( $\bar{K}{\;}_{i}$ value) as the ordinate. The relationship diagram of each factor level and the average comprehensive hygroscopic moisture guide index is shown in Figure 6. The figure illustrates the varying degrees of influence of each level in the upper yarn configuration of factor A on the moisture absorption and humidity conduction of the fabrics. The ranking is as follows: A_2_ > A_3_ > A_4_ > A_5_ > A_1_. This indicates that when the proportion of UHMWPE filament in the upper yarn configuration is 75%, the fabric exhibits the best moisture absorption and humidity conduction. Conversely, when the upper yarn is entirely polyamide, the fabric demonstrates the worst moisture absorption and humidity conduction. This is because the UHMWPE filament has poor moisture absorption properties, while the polyamide fiber has better moisture absorption properties. When the upper yarn contains UHMWPE, the fabric can form a structure with one side being hydrophobic and the other side being hydrophilic, achieving a unidirectional moisture absorption and humidity conduction effect. However, when the upper yarn is filled with UHMWPE filament, water is not easily absorbed. Conversely, when the upper yarn contains a significant amount of polyamide, the water is not easily conducted after absorption, thus preventing the achievement of effective unidirectional moisture absorption and humidity conduction. The order of influence of each level in the organizational structure of factor B on the moisture absorption and humidity conduction of the fabrics is as follows: B_5_ > B_4_ > B_2_ > B_3_ > B_1_. This indicates that the moisture absorption and humidity conductivity of the fabric formed by the 2 separate 2 float wire organization is better, while the flat needle plating organization is the worst. This may be because the fabric formed by the 2 separate 2 float wire organization is denser, with more interweaving points, and has more water absorption and conduction channels, resulting in better moisture absorption and humidity conductivity.

According to the orthogonal analysis results above, a correlation analysis was conducted between the comprehensive hygroscopic moisture guide index *C* of the fabric and the fabric specification parameters, as well as other performance indexes. The results are presented in Table 11. It is evident that the comprehensive hygroscopic moisture conductivity index *C* of fabric exhibits a significant positive correlation (0.5 < |r| ≤ 0.8) with the moisture permeability of the fabric.

### 3.4. Thermal Property

The properties of fibers (thermal conductivity) and fabric structures (mass per unit area and thickness, etc.) are the main factors that affect the thermal properties of fabrics [47]. The orthogonal test results for the thermal property test in Table 4 are presented in Table 12. It is evident that the arrangement of the upper yarn in the fabric had a significant impact on the thermal properties of the sample. In addition, as the thermal resistance and warmth retention ratio decrease, the heat transfer coefficient increases, leading to better thermal performance of the sample. Therefore, the best weaving scheme to achieve the desired thermal properties of the fabric is A_1_B_4_.

**Figure 7:**
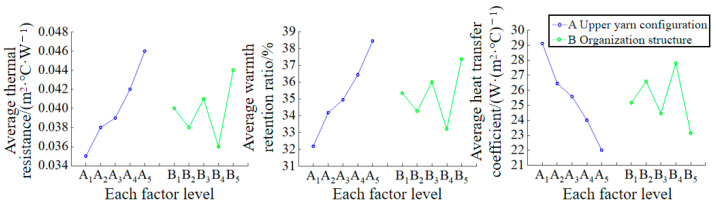
Figure of the relationship between the level of each factor and the average thermal resistance, warmth retention ratio and heat transfer coefficient of the sample.

To intuitively study the influence of the two factors and five levels of the design on the thermal properties of the sample, each factor level was used as the horizontal coordinate. The average thermal resistance, average warmth retention ratio, and average heat transfer coefficient of the fabric at each factor level ( $\bar{K}{\;}_{i}$ value) were used as the longitudinal coordinates. Subsequently, the parameter relationship diagram of each factor level and average thermal properties was drawn, as shown in Figure 7. It can be observed from the figure that the influence of each level in the upper yarn configuration of factor A on the heat transfer coefficient of the fabric is ranked as follows: A_1_ > A_2_ > A_3_ > A_4_ > A_5_. The influence on thermal resistance and warmth retention ratio of fabrics is opposite to the heat transfer coefficient. The results indicated that as the proportion of UHMWPE filament in the upper yarn configuration increased, the thermal resistance and warmth retention ratio of the fabric decreased, the heat transfer coefficient increased, and the thermal conductivity improved. This is because, under the same organizational structure, UHMWPE filament has a higher thermal conductivity than polyamide filament. By increasing the proportion of UHMWPE filament, the fabric thickness decreases, enhancing heat transfer ability, shortening the heat transfer path, and improving the fabric’s thermal conductivity. The order of influence of each level in the organizational structure of factor B on the heat transfer coefficient of the fabrics is as follows: B_4_ > B_2_ > B_1_ > B_3_ > B_5_. The degree of influence on thermal resistance and warmth retention ratio of fabrics is opposite to the heat transfer coefficient. The thermal conductivity of the fabric formed by the tuck plating technique is the highest, followed by the flat needle plating organization, and the float wire plating organization is the lowest. This is because, under the same yarn preparation, the fabric formed by the tuck plating organization is thinner, has more open space, and allows heat to pass through more easily than the fabric formed by the other two types of organizational structures.

According to the orthogonal analysis results above, the thermal resistance, warmth retention ratio, and heat transfer coefficient of the fabric were found to be correlated with the mass per unit area, fabric thickness, the proportion of UHMWPE filament in the upper yarn configuration, air permeability, and moisture permeability, respectively. These results are presented in Table 13. It is evident that the thermal resistance, warmth retention ratio of the fabric, mass per unit area, thickness, the proportion of UHMWPE filament in the upper yarn configuration, and air permeability all exhibit significant correlation (0.5 < |r| ≤ 0.8) or high correlation (0.8 < |r| ≤ 1). They are positively correlated with the mass per unit area and thickness, and negatively correlated with the proportion of UHMWPE filament in the upper yarn configuration and air permeability. The greater the mass per unit area and thickness of the UHMWPE filament in the upper yarn configuration, the lower the air permeability of the fabric. This results in higher thermal resistance, better warmth retention, and improved thermal insulation performance. The heat transfer coefficient of the fabric showed a significant correlation (0.5 < |r| ≤ 0.8) or high correlation (0.8 < |r| ≤ 1) with the mass per unit area, thickness, the proportion of UHMWPE filament in the upper yarn configuration, and air permeability. It was negatively correlated with the mass per unit area and thickness, and positively correlated with the proportion of UHMWPE filament in the upper yarn configuration. The results indicated that the fabric with a smaller mass per unit area, thinner thickness, a larger proportion of UHMWPE filament in the upper yarn configuration, and higher air permeability exhibited a higher heat transfer coefficient and better diathermy.

### 3.5. Contact Cool Feeling Property

There are numerous factors that affect the contact cool feeling property of fabrics, including fiber materials, fabric structure, and more [48]. According to GB/T 35263-2017, if the contact cooling coefficient (q_max_) of the fabric is 0.15 or higher, the fabric exhibits good contact cooling sensitivity. From Table 4 we can see that the contact cooling coefficient (q_max_) of all 25 designed samples exceeds 0.15, indicating that the contact cooling sensitivity of all samples is good. The orthogonal test results for the contact cool feeling property test in Table 4 are presented in Table 14. It can be seen that the upper yarn configuration of the fabric had a significant impact on the sample’s contact cool feeling property. In addition, it can also be seen from the table that the best weaving scheme to achieve the cool feeling property by making the fabric contact is A_1_B_5_.

**Figure 8:**
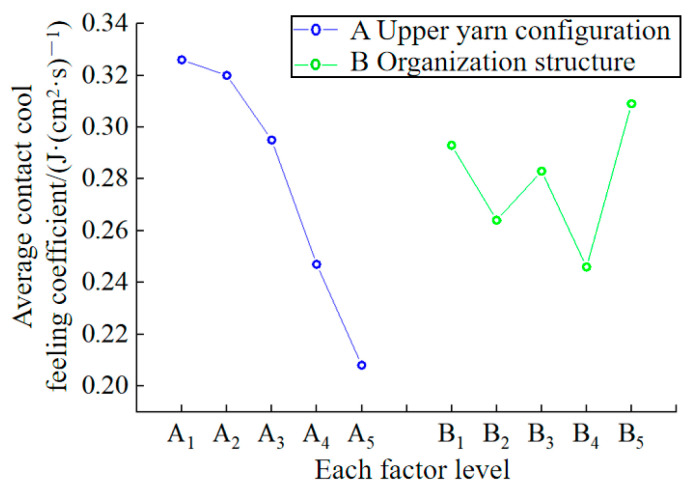
Figure of the relationship between the level of each factor and the average contact cool feeling coefficient of the sample.

In order to study the influence of the two factors and five levels of the design on the contact cool feeling property of the sample in a more intuitive manner, each factor level was used as the horizontal coordinate. The average contact cool feeling coefficient ( $\bar{K}{\;}_{i}$ value) of the fabric under each factor level was used as the longitudinal coordinate. A relationship diagram between the level of each factor and the average contact cool feeling coefficient was then drawn, as shown in Figure 8. The figure illustrates the varying degrees of influence of each level in the upper yarn configuration of factor A on the contact cool feeling coefficient of the fabrics, with the ranking as follows: A_1_ > A_2_ > A_3_ > A_4_ > A_5_. This indicates that as the proportion of UHMWPE filament in the upper yarn decreases, the contact cool feeling coefficient of the fabric also decreases, leading to a deterioration in the contact cool feeling property. This is because, under the same organizational structure, the thermal conductivity of UHMWPE fiber is higher than that of polyamide. The higher the UHMWPE fiber content, the greater the thermal conductivity of the fabric and the stronger the sensation of coolness. The influence degree of each level in the organizational structure of factor B on the contact cool feeling coefficient of the fabrics is as follows: B_5_ > B_1_ > B_3_ > B_2_ > B_4_. This indicates that the contact cool feeling property of the fabric formed by the float wire plating organization and the flat needle plating organization weave is better than that of the tuck plating organization. The contact area between the fabric and the skin is larger when using the float wire plating and flat needle plating techniques compared to the tuck plating organization under the same yarn preparation. This results in a stronger feeling of coolness due to increased heat conduction channels.

Based on the orthogonal analysis results above, a correlation analysis was conducted between the fabric’s contact cool feeling coefficient and its specification parameters and other performance indicators, as presented in Table 15. It is evident that the contact cool feeling coefficient of the fabric shows no correlation (0 < |r| ≤ 0.3) with the mass per unit area, thickness, air permeability, moisture permeability, and comprehensive hygroscopic moisture guide index of the fabric. However, it is highly correlated (0.8 < |r| ≤ 1) with the proportion of UHMWPE filament in the upper yarn configuration, and the correlation is positive. It is indicated that the fabric with a higher proportion of UHMWPE filament in the upper yarn configuration exhibits a larger contact cool feeling coefficient and better contact cool feeling properties. There is a low positive correlation (0.3 < |r| ≤ 0.5) between the heat transfer coefficient of the fabric and the contact cool feeling coefficient, suggesting that fabrics with a higher heat transfer coefficient provide a better cool feeling.

## 4. Conclusions

To investigate the impact of UHMWPE fiber content and knitting structure on the heat and humidity comfort and contact cool feeling property of fabrics, five types of upper yarns with varying proportions were designed using UHMWPE filament and polyamide filament. The UHMWPE fiber proportions were 100%, 75%, 50%, 25%, and 0%, respectively. There are five types of knitting structures based on the plating stitch, including flat needle plating, single needle single column set circle with added yarn, 1 separate 1 float wire, double needle and double column set circle with added yarn, and 2 separate 2 float wire. These structures can produce 25 different types of knitted fabrics on the machine. The air permeability, moisture permeability, moisture absorption and humidity conduction, thermal property, and contact cool feeling property of the fabrics were investigated using orthogonal analysis and correlation analysis. The results indicate that for the same yarn configuration, the samples exhibit good air permeability, moisture permeability, and heat conductivity. Additionally, the samples with a higher proportion of UHMWPE filament in the veil demonstrate better moisture absorption and contact sensitivity. In the case of the same UHMWPE and polyamide composition, the added tissue shows improved moisture permeability and moisture absorption, as well as enhanced air permeability and contact conductivity with a higher proportion of UHMWPE filament in the veil. In general, the knitted fabric made by using UHMWPE fiber in a reasonable manner exhibits excellent heat and humidity comfort performance, offering a new way for the development of cool and comfortable fabrics.

## Figures and Tables

**Table 1:** Thermal conductivity of common fibers.

Fibre Type	Thermal Conductivity/(W·(m·°C)^−1^)	Fibre Type	Thermal Conductivity/(W·(m·°C)^−1^)	Fibre Type	Thermal Conductivity/(W·(m·°C)^−1^)
silk	0.050~0.055	viscose fibre	0.055~0.071	polypropylene	0.221~0.302
cotton	0.071~0.073	acetate fibre	0.05	polyvinyl chloride	0.042
wool	0.052~0.055	polyester	0.084	water (non-fiber)	0.697
polyamide	0.244~0.337	acrylic	0.051	air (non-fiber)	0.026

**Table 2:** Factor-level table of experimental samples.

	Factor	A: Upper Yarn Configuration (Upper Yarn Feeding Method)	B: Organizational Structure
Level			
1		8U0N (8 roads UHMWPE + 0 road polyamide)	a (flat needle plating organization)
2		6U2N (6 roads UHMWPE + 2 roads polyamide)	b (single needle single column set circle add yarn organization)
3		4U4N (4 roads UHMWPE + 4 roads polyamide)	c (1 separate 1 float wire organization)
4		2U6N (2 roads UHMWPE + 6 roads polyamide)	d (double needle and double column set circle to add yarn organization)
5		0U8N (0 road UHMWPE + 8 roads polyamide)	e (2 separate 2 float wire organization)

**Table 3:** Basic specification parameters of the experimental samples.

Sample Number	A: Upper Yarn Configuration	B: Organizational Structure	Mass per Unit Area /(g·m^−2^)	Course Density /(wale·cm^−1^)	Wale Density /(course·cm^−1^)	Thickness /mm
1#	A_1_: 8U0N	B_1_: a	211	17	25	0.9
2#	A_1_: 8U0N	B_2_: b	196	17	26	0.93
3#	A_1_: 8U0N	B_3_: c	250	17	24	0.96
4#	A_1_: 8U0N	B_4_: d	187	17	26	0.93
5#	A_1_: 8U0N	B_5_: e	289	17	26	1.1
6#	A_2_: 6U2N	B_1_: a	211	17	24	0.92
7#	A_2_: 6U2N	B_2_: b	197	17	26	0.95
8#	A_2_: 6U2N	B_3_: c	254	17	25	0.99
9#	A_2_: 6U2N	B_4_: d	195	17	26	0.95
10#	A_2_: 6U2N	B_5_: e	292	17	25	1.11
11#	A_3_: 4U4N	B_1_: a	219	17	26	0.95
12#	A_3_: 4U4N	B_2_: b	198	17	27	0.96
13#	A_3_: 4U4N	B_3_: c	266	17	25	1.01
14#	A_3_: 4U4N	B_4_: d	198	17	26	0.96
15#	A_3_: 4U4N	B_5_: e	294	17	26	1.11
16#	A_4_: 2U6N	B_1_: a	220	17	26	0.96
17#	A_4_: 2U6N	B_2_: b	200	17	26	0.96
18#	A_4_: 2U6N	B_3_: c	267	18	23	1.01
19#	A_4_: 2U6N	B_4_: d	199	17	26	0.97
20#	A_4_: 2U6N	B_5_: e	295	18	27	1.12
21#	A_5_: 0U8N	B_1_: a	237	17	26	0.97
22#	A_5_: 0U8N	B_2_: b	216	17	28	0.97
23#	A_5_: 0U8N	B_3_: c	283	17	26	1.01
24#	A_5_: 0U8N	B_4_: d	216	17	29	0.98
25#	A_5_: 0U8N	B_5_: e	308	19	27	1.14

**Table 4:** Performance test results of the experimental samples.

Sample Number	*Q* /(mm·s^−1^)	*WVT* /(g·(m^2^·24 h)^−1^)	*T*1 /s	*T*2 /s	*A*1 /(%·s^−1^)	*A*2 /(%·s^−1^)	*O*	*R_t_* /(m^2^·°C·W^−1^)	*W_t_*	*K_t_* /(W·(m^2^·°C)^−1^)	*q*_max_ /(J·(cm^2^·s)^−1^)
1#	445.24	2942.756	6.7	5.2	43.2	48.6	100.31	0.0372	33.84	26.81	0.340
2#	533.61	3036.042	7.2	15.6	45.0	59.7	100.72	0.0303	29.37	32.98	0.319
3#	260.32	2825.795	6.1	3.6	38.3	39.0	111.44	0.0377	34.13	26.46	0.324
4#	557.20	3265.018	6.1	7.8	42.0	45.1	169.60	0.0303	29.36	33.00	0.300
5#	218.96	2756.184	5.6	19.0	48.3	857.0	194.61	0.0379	34.23	26.35	0.346
6#	364.72	3431.095	4.6	11.8	73.6	141.2	121.50	0.0378	34.17	26.42	0.327
7#	530.20	3451.590	2.1	2.1	73.6	80.9	240.39	0.0377	34.14	26.46	0.318
8#	246.42	3061.484	5.2	5.2	38.0	41.7	255.30	0.0395	35.15	25.30	0.322
9#	549.33	3756.890	5.2	11.3	55.5	109.7	285.30	0.0345	32.14	28.95	0.296
10#	210.84	2968.198	3.1	2.6	90.8	103.0	372.81	0.0398	35.32	25.11	0.338
11#	336.66	3926.148	2.6	4.1	59.2	56.9	−117.59	0.0392	35.03	25.44	0.325
12#	525.78	4096.113	2.6	3.6	55.7	57.1	−48.91	0.0383	34.47	26.07	0.254
13#	208.80	3578.799	3.6	5.1	52.5	63.5	19.94	0.0399	35.38	25.05	0.318
14#	527.52	4443.816	2.6	4.1	57.6	66.3	118.28	0.0359	33.01	27.83	0.240
15#	186.22	3248.057	4.1	2.6	51.6	55.9	186.02	0.0425	36.85	23.50	0.336
16#	299.32	3816.254	3.1	4.1	62.8	54.8	−120.16	0.0401	35.54	24.88	0.260
17#	512.10	3875.618	2.6	5.1	58.0	63.6	32.72	0.0399	35.40	25.03	0.229
18#	201.50	3256.537	2.6	7.5	49.8	57.3	28.01	0.0415	36.31	24.05	0.238
19#	513.72	3962.898	2.6	4.1	58.7	72.2	142.86	0.0395	35.18	25.27	0.207
20#	175.62	3146.290	4.6	5.2	54.7	59.2	140.25	0.0480	39.74	20.79	0.300
21#	232.96	2628.975	5.2	7.2	62.9	59.6	−209.31	0.0448	38.11	22.27	0.215
22#	305.86	3349.823	2.6	12.3	54.2	111.1	−111.38	0.0446	37.99	22.38	0.202
23#	156.42	2527.208	3.1	7.2	51.1	65.5	9.89	0.0466	39.03	21.42	0.211
24#	326.74	3375.265	3.6	5.7	51.8	63.2	46.72	0.0416	36.37	23.99	0.186
25#	156.38	2510.247	4.6	5.7	44.0	56.0	141.31	0.0500	40.71	19.97	0.224

**Table 5:** Standard deviation values of the various performance test results of the fabrics.

Sample Number	*Q* /(mm·s^−1^)	*WVT* /(g·(m^2^·24 h)^−1^)	*T*1 /s	*T*2 /s	*A*1 /(%·s^−1^)	*A*2 /(%·s^−1^)	*O*	*K_t_* /(W·(m^2^·°C)^−1^)	*q*_max_ /(J·(cm^2^·s)^−1^)
1#	13.683	47.192	0.122	0.255	0.158	0.265	0.383	0.557	0.007
2#	14.763	37.598	0.158	0.255	0.187	0.367	0.315	0.191	0.005
3#	15.049	44.281	0.071	0.187	0.255	0.332	0.389	0.361	0.004
4#	20.141	5.553	0.187	0.122	0.212	0.308	0.828	0.082	0.007
5#	10.556	38.825	0.158	0.212	0.381	1.340	0.587	0.164	0.003
6#	6.950	22.351	0.187	0.255	0.292	0.552	0.433	0.131	0.005
7#	14.370	15.265	0.187	0.100	0.122	0.308	0.429	0.115	0.003
8#	12.469	4.151	0.158	0.187	0.122	0.430	0.446	0.433	0.004
9#	13.983	25.552	0.173	0.224	0.122	0.367	0.819	0.957	0.005
10#	5.752	45.133	0.158	0.187	0.122	0.453	0.617	0.085	0.004
11#	8.094	23.394	0.158	0.122	0.235	0.354	0.888	0.185	0.003
12#	20.588	23.246	0.122	0.158	0.339	0.400	0.450	0.070	0.004
13#	3.816	27.531	0.212	0.122	0.245	0.604	0.471	0.082	0.002
14#	7.540	11.778	0.122	0.187	0.339	0.474	0.336	0.233	0.002
15#	8.912	13.718	0.122	0.224	0.418	0.339	0.386	0.098	0.003
16#	7.837	15.625	0.122	0.122	0.245	0.510	0.664	0.174	0.002
17#	13.012	6.276	0.122	0.071	0.274	0.406	0.435	0.151	0.002
18#	8.430	17.190	0.187	0.122	0.418	0.675	0.375	0.131	0.002
19#	9.816	7.163	0.122	0.173	0.187	0.608	0.361	0.069	0.004
20#	8.844	25.152	0.187	0.187	0.339	0.561	0.522	0.053	0.002
21#	8.967	25.215	0.235	0.122	0.381	0.524	0.486	0.052	0.004
22#	9.618	9.457	0.212	0.255	0.308	0.604	0.622	0.017	0.003
23#	5.752	22.961	0.100	0.141	0.255	0.367	0.434	0.062	0.003
24#	5.044	7.084	0.212	0.122	0.381	0.583	0.542	0.053	0.001
25#	8.932	12.139	0.071	0.122	0.339	0.604	0.485	0.111	0.004

**Table 6:** Analysis results of orthogonal test of fabric air permeability.

Factor	$\bar{K}{\;}_{1}$	$\bar{K}{\;}_{2}$	$\bar{K}{\;}_{3}$	$\bar{K}{\;}_{4}$	$\bar{K}{\;}_{5}$	R	The Best Level of Factors
A	403.066	380.302	356.996	340.452	235.672	167.394	A_1_
B	335.780	481.510	214.692	494.902	189.604	305.298	B_4_

Note: $\bar{K}{\;}_{i}$ represents the mean of the sum of experimental results for this factor at level i, and is used to calculate the R-value, where i is 1,2, …, 5; The R-value is the range value, which is the largest $\bar{K}{\;}_{i}$ minus the smallest $\bar{K}{\;}_{i}$ , and is used to rank the primary and secondary factors.

**Table 7:** The correlation between fabric air permeability and fabric specification parameters.

Fabric Specification Parameters	Mass per Unit Area	Thickness	*P_U_*
Correlation coefficient r	−0.978	−0.847	0.439
Significant difference *p* value	0.000 ***	0.000 ***	0.028 **

Note: *** and ** represent 1% and 5% significance levels respectively.

**Table 8:** Analysis results of fabric moisture permeability orthogonal test.

Factor	$\bar{K}{\;}_{1}$	$\bar{K}{\;}_{2}$	$\bar{K}{\;}_{3}$	$\bar{K}{\;}_{4}$	$\bar{K}{\;}_{5}$	R	The Best Level of Factors
A	2965.159	3333.851	3858.587	3611.519	2878.304	980.283	A_3_
B	3349.046	3561.837	3049.965	3760.777	2925.795	834.982	B_4_

**Table 9:** Correlation between fabric moisture permeability and fabric specification parameters and other performance indicators.

Fabric Specification Parameters	Mass per Unit Area	Thickness	*P_U_*	Air Permeability
Correlation coefficient r	−0.576	−0.388	−0.063	0.58
Significant difference *p* value	0.003 ***	0.056 *	0.766	0.002 ***

Note: *** and * represent 1% and 10% significance levels respectively.

**Table 10:** Analysis results of orthogonal test of fabric moisture absorption.

	*T*1		*T*2		*A*1		*A*2		*O*		*C*	
	A	B	A	B	A	B	A	B	A	B	A	B
$\bar{K}{\;}_{1}$	0.169	0.541	0.518	0.741	0.102	0.423	0.209	0.041	0.592	0.282	1.589	2.028
$\bar{K}{\;}_{2}$	0.620	0.741	0.734	0.666	0.536	0.366	0.069	0.043	0.798	0.433	2.756	2.249
$\bar{K}{\;}_{3}$	0.804	0.604	0.893	0.786	0.328	0.150	0.026	0.018	0.414	0.505	2.465	2.063
$\bar{K}{\;}_{4}$	0.804	0.624	0.817	0.734	0.356	0.286	0.027	0.039	0.436	0.622	2.440	2.305
$\bar{K}{\;}_{5}$	0.663	0.549	0.673	0.709	0.280	0.377	0.039	0.229	0.317	0.715	1.973	2.578
The best level of factors											A_2_	B_5_

**Table 11:** The correlation between comprehensive hygroscopic moisture guide index of fabrics and fabric specifications and other performance indexes.

Fabric Specification Parameters	Mass per Unit Area	Thickness	*P_U_*	Air Permeability	Moisture Permeability
Correlation coefficient r	0.035	0.184	−0.161	0.019	0.595
Significant difference *p* value	0.868	0.378	0.443	0.927	0.002 ***

Note: *** represents 1% significance level.

**Table 12:** Results of orthogonal test on thermal properties of fabrics.

	*R_t_*/(m^2^·°C·W^−1^)		*W_t_*		*K_t_*/(W·(m^2^·°C)^−1^)	
	A	B	A	B	A	B
$\bar{K}{\;}_{1}$	0.035	0.040	32.186	35.338	29.120	25.164
$\bar{K}{\;}_{2}$	0.038	0.038	34.184	34.274	26.448	26.584
$\bar{K}{\;}_{3}$	0.039	0.041	34.948	36.000	25.578	24.456
$\bar{K}{\;}_{4}$	0.042	0.036	36.434	33.212	24.004	27.808
$\bar{K}{\;}_{5}$	0.046	0.044	38.442	37.370	22.006	23.144
R	0.011	0.007	6.256	4.158	7.114	4.664
The best level of factors	For the thermal conductivity of the sample, the smaller the value of thermal resistance and warmth retention ratio, the larger the value of heat transfer coefficient, the better the thermal conductivity. Therefore, the best factor level of the thermal properties of the sample is A_1_B_4_					

**Table 13:** The correlation of fabric thermal properties with fabric specifications and other properties.

Fabric Specifications and Performance Indicators		Mass per Unit Area	Thickness	*P_U_*	Air Permeability	Moisture Permeability	*C*
*R_t_*	Correlation coefficient r	0.733	0.761	−0.848	−0.805	−0.295	0.088
	Significant difference *p* value	0.000 ***	0.000 ***	0.000 ***	0.000 ***	0.152	0.677
*W_t_*	Correlation coefficient r	0.721	0.754	−0.855	−0.793	−0.285	0.103
	Significant difference *p* value	0.000 ***	0.000 ***	0.000 ***	0.000 ***	0.167	0.624
*K_t_*	Correlation coefficient r	−0.726	−0.755	0.853	0.797	0.290	−0.096
	Significant difference *p* value	0.000 ***	0.000 ***	0.000 ***	0.000 ***	0.160	0.649

Note: *** represents 1% significance level.

**Table 14:** Results of orthogonal test of fabric contact cool feeling property.

Factor	$\bar{K}{\;}_{1}$	$\bar{K}{\;}_{2}$	$\bar{K}{\;}_{3}$	$\bar{K}{\;}_{4}$	$\bar{K}{\;}_{5}$	R	The Best Level of Factors
A	0.326	0.32	0.295	0.247	0.208	0.118	A_1_
B	0.293	0.264	0.283	0.246	0.309	0.063	B_5_

**Table 15:** The correlation of fabric contact cool feeling coefficient with fabric specifications and other properties.

Fabric Specification	Mass per Unit Area	Thickness	*P_U_*	Air Permeability	Moisture Permeability	*C*	Heat Transfer Coefficient
Correlation coefficient r	0.146	−0.164	0.816	−0.007	−0.199	0.012	0.476
Significant difference *p* value	0.485	0.434	0.000 ***	0.974	0.340	0.956	0.016 **

Note: *** and ** represent 1% and 5% significance levels respectively.

## Data Availability

The data are contained within the article.

## References

[1] Ji B.L., Wang B.J., Mao Z.P. (2022). Key technologies supporting low-carbon emissions in dyeing and finishing of textiles. J. Text. Res..

[2] Yao P.P. (2019). Research on the cool-feeling fabrics. China Fiber Insp..

[3] Bhuiyan M.A.R., Wang L., Shaid A., Jahan I., Shanks R.A. (2020). Silica aerogel-integrated nonwoven protective fabrics for chemical and thermal protection and thermophysiological wear comfort. J. Mater. Sci..

[4] Farooq A.S., Zhang P. (2021). Fundamentals, materials and strategies for personal thermal management by next-generation textiles. Compos. Part A Appl. Sci. Manuf..

[5] Zeng S.N., Hu J.Y., Zhang M.N., Xiang Y.Z., Wu J.W., Su M.Y., Zhang Y.Q., Shen M., Hong P., Huang Z.L. (2022). Cooling textiles for personal thermal management. Chin. Sci. Bull..

[6] Chen J. (2020). Innovative development and future trend of functional home textiles. China Text. Lead..

[7] Li Y.H., Liu Y., Jia Y.H., Bai Z.H., Liu L., Zhang R.Y., Du L.X. (2023). Research status and progress of cool functional textiles. Wool Text. J..

[8] Zhou Z.Y., Zhang X.Q. (2021). Research progress on development of cool-feeling fibers and its detection method. Synth. Fiber China.

[9] Yang Y., Yu X., Wang X.G., Sun Y., Zhang P., Liu X. (2020). Effect of jade nanoparticle content and twist of cool-touch polyester filaments on comfort performance of knitted fabrics. Text. Res. J..

[10] Li J.S., Chen H.H., Guan F.W., Kun H. (2022). Application and development prospect of jade fiber in summer knitted garment. Front. Art Res..

[11] Yue P.F., Zhang Y.X., Sheng C.H., Zhang X.Q. (2020). The development of cool functional woven fabric made of mica fiber and coolmax fiber. Synth. Fiber China.

[12] Zhang J.Y. (2014). Study on Test and Evaluation for Absorption and Quick-Drying Properties of Shaped Fibers and the Fabric. Ph.D. Thesis.

[13] Qin Q.E., Zhou T., Wang M., Li L., Chen N. (2023). Structure evolution and performance of poly (vinyl alcohol) fibers with controllable cross-section fabricated using a combination of melt-spinning and stretching. Polym. Test..

[14] Yang Y., Yu X., Wang X.G., Liu X., Zhang P. (2021). Thermal comfort properties of cool-touch nylon and common nylon knitted fabrics with different fibre fineness and cross-section. Ind. Textila.

[15] Liao S.H., Wu H., Shen L.P., Ling Z.C. (2020). Effect of fabric weave structure on properties of graphene/wool worsted shirt fabric. Synth. Fiber China.

[16] Zou W.L., Zhu B.L., Xu R.C. (2012). Research of Outlast modified acrylic fiber blended fabric temperature-adjusting property. Cotton Text. Technol..

[17] Qian J., Xie T., Chen L.Q., Li Z., Guo N., Fu S., Zhang P. (2022). Effect of knitting structure and polyethylene content on thermal-wet comfort and cooling properties of polyethylene/polyester fabrics. Fibers Polym..

[18] Jia Y.H., Li Y.X. (2021). Development and practice of a cool cotton fabric. China Text. Lead..

[19] Wang G. (2015). Development and Performance Research of Cool Nylon Filament Knitted Products. Ph.D. Thesis.

[20] Chen Y.P. (2016). Performance Research and Comprehensive Evaluation of Jade Fiber Summer Sports Knitted Fabrics. Ph.D. Thesis.

[21] Bait S.H., Shrivastava N., Behera J., Ramakrishnan V., Dayal A., Jadhav G. (2019). Development of sportswear with enhanced moisture management properties using cotton and regenerated cellulosic fibres. Indian J. Fibre Text. Res..

[22] Xie T., Qian J., Zhang P.H. (2021). Application and prospect of the cool feeling polyethylene fiber in cool textiles. Tech. Text..

[23] Alberghini M., Hong S., Lozano L.M., Korolovych V., Huang Y., Signorato F., Zandavi S.H., Fucetola C., Uluturk I., Tolstorukov M.Y. (2021). Sustainable polyethylene fabrics with engineered moisture transport for passive cooling. Nat. Sustain..

[24] Shen X.X. (2017). Study on the Technology and Performance of Viscose/UHMWPE Yarn Bed Fabric. Ph.D. Thesis.

[25] Zhou L.Y., Wang Y.P. (2011). Modern Clothing Materials Science.

[26] Yuxiu B., Hong X., Yueping W., Chao S., Weijing Y. (2020). Influence of fabric parameters on thermal conductivity of UHMWPE interwoven fabric. Wool Text. J..

[27] Zhang Q.S. (2021). Research on Cool Feeling Fabric. Ph.D. Thesis.

[28] Song G.L. (2013). Review of the circular seamless underwear knitting machines on the 2016 china international textile machinery exhibition-ITMA Asia. Knitt. Ind..

[29] Shen H., Shao X.H. (2007). Equipment and process of producing seamless underwear. Prog. Text. Sci. Technol..

[30] Yin A. (2022). Development of Knitted Woolen Sports Fabric Based on Plasma Technology. Ph.D. Thesis.

[31] Mavruz S., Ogulata R.T. (2009). Investigation and statistical prediction of air permeability of cotton knitted fabrics. Tekst. Konfeksiyon.

[32] Zhang Y., Ma X.A. (2013). Comparison of two calculation methods for textile air-permeability. Knitt. Ind..

[33] (1997). Textiles—Determination of the Permeability of Fabrics to Air.

[34] Huang J.H., Qian X.M. (2008). Comparison of test methods for measuring water vapor permeability of fabrics. Text. Res. J..

[35] (2009). Textiles—Test Method for Water-Vapor Transmission of Fabrics—Part 1: Desiccant Method.

[36] Zhao K., Wang Y., Wang W., Yu D. (2018). Moisture absorption, perspiration and thermal conductive polyester fabric prepared by thiol-ene click chemistry with reduced graphene oxide finishing agent. J. Mater. Sci..

[37] (2009). Textiles—Evaluation of Absorption and Quick-Drying Part 2: Method for Moisture Management Tests.

[38] Afzal A., Ahmad S., Rasheed A., Ahmad F., Iftikhar F., Nawab Y. (2017). Influence of fabric parameters on thermal comfort performance of double layer knitted interlock fabrics. Autex Res. J..

[39] (2008). Textiles—Physiological Effects—Measurement of Thermal and Water-Vapor Resistance under Steady-State Conditions.

[40] Ni Q.M. (2021). Study on the Preparation and Evaluation of the Cool-Feel Knitted Fabric. Ph.D. Thesis.

[41] (2017). Textiles—Testing and Evaluation for Instant Contact Cool Feeling.

[42] Muraliene L., Mikucioniene D. (2020). Influence of structure and stretch on air permeability of compression knits. Int. J. Cloth. Sci. Technol..

[43] Cui Y.Y. (2023). Discussion on the test methods and influencing factors of moisture permeability of textile fabrics. Text. Test. Stand..

[44] Manohari B.G., Kannappan J., Kandhavadivu P., Ramachandran T., Liu C. (2011). Liquid moisture transmission behavior of microfiber blended knitted fabrics. Melliand China.

[45] Zhang F.F., Wang W.A., Jing Z.Y., Jiang X.T., Xin H. (2022). Research status and progress of unidirectional water-transport fabrics. Wool Text. J..

[46] Wei C.Y., Cui Y.Z., Jiang F.Q., Fu C.L. (2011). The influence of fabric structure on moisture absorbency and sweat transport. Shanghai Text. Sci. Technol..

[47] Qian J., Xie T., Zhang P.H., Fu S.J. (2022). Thermal and moisture comfort performance of polyethylene knitted fabric. J. Text. Res..

[48] Li X.M., Wang S.H., Li Y.Q., Jin X., Ma L., Tian W., Zhu C. (2023). Evaluation model of fabric transient cooling sensation based on multiple stepwise regression analysis. J. Eng. Fibers Fabr..

